# Time to first birth and its predictors among reproductive-age women in Ethiopia: inverse Weibull gamma shared frailty model

**DOI:** 10.1186/s12905-021-01254-z

**Published:** 2021-03-19

**Authors:** Reta Dewau, Fantahun Ayenew Mekonnen, Wullo Sisay Seretew

**Affiliations:** 1 Department of Epidemiology and Biostatistics, School of Public Health, College of Medicine and Health Science, Wollo University, Dessie, Ethiopia; 2 Department of Epidemiology and Biostatistics, Institute of Public Health, College of Medicine and Health Sciences, University of Gondar, Gondar, Ethiopia

**Keywords:** Time to first birth, Predictors, Reproductive age-women, Ethiopia

## Abstract

**Background:**

High maternal and child death with high fertility rate have been reported in Ethiopia. Extreme age at first birth is linked with both maternal and child morbidity and mortality. However, literatures showed there were limited studies on the timing of the first birth and its predictors in the area so far. Therefore, determining the time to first birth and its predictors will help to design strategies to improve maternal and child survival.

**Methods:**

A community-based cross-sectional study was conducted among reproductive-age women in Ethiopia using the Ethiopian demographic health survey, 2016 data. Stratified two-stage cluster sampling technique was used for sampling. The Kaplan–Meier method was used to estimate time to first birth. Inverse Weibull gamma shared frailty model applied to model the data at 95% confidence interval (CI), adjusted hazard ratio (AHR) and median hazard ratio (MHR) were reported as effect size. Proportional hazard assumption checked using Schoenfeld residual test. Information Criteria were applied to select a parsimonious model. Stratified analysis performed for the interaction terms and statistical significance was declared at *p* value < 0.05.

**Results:**

The overall median age at first birth was found to be 20 years (IQR, 16–24 years). The independent predictors of time to first birth were: married 15–17 years (AHR = 2.33, 95% CI 2.08–2.63), secondary education level (AHR = 0.84, 95% CI 0.78–0.96), higher education level (AHR = 0.75, 95% CI 0.65–0.85), intercourse before 15 years in the married stratum (AHR = 23.81, 95% CI 22.22–25.64), intercourse 15–17 years in married stratum (AHR = 5.56, 95% CI 5.26–5.88), spousal age difference (AHR = 1.11, 95% CI 1.05–1.16),and use of contraceptives (AHR = 0.91, 95% CI 0.86–0.97). The median increase in the hazard of early childbirth in a cluster with higher early childbirth is 16% (MHR = 1.16, 95% CI 1.13–1.20) than low risk clusters adjusting for other factors.

**Conclusion:**

In this study, first birth was found to be at an early age. Early age at first marriage, at first sexual intercourse and their interaction, high spousal age difference, being Muslim were found to increase early motherhood. Conversely, living in the most urban region, secondary and higher women education were identified to delay the first birth. Investing on women education and protecting them from early marriage is required to optimize time to first birth. The contextual differences in time to first birth are an important finding which requires more study and interventions.

## Background

Age at first birth refers to the age of a mother when she gave birth to her first child [1, 2]. Attaining the first child is one of the most important events in a woman's life. It indicates the beginning of the intensive responsibilities of maternity and childcare [3].

Roughly 1 in 10 childbirths contributed by young mothers worldwide and of these, developing countries accounting 95% of the share [4]. Girls under 15 years account for 2 million (27%) of the 7.3 million births that occur to adolescent girls below s18 years in developing countries [5].

When a woman became pregnant in the adolescent period, her present and future life rarely became for better [5, 6]. It results in cessation of education, joblessness, deprived maternal and child health outcomes, an numerous children per women, gender inequity, destitution of adolescent mothers and their families and the communities at large [2, 5, 7–13]. Worldwide over half a million (500,000) women aged 15–49 years die annually from preventable pregnancy-related complications [14]. Furthermore, girls under 15 years are five times at higher risk of death and those 15–19 years are twice more likely to die than women aged 20–24 years in pregnancy or childbirth [15, 16]. Complications from Pregnancy and childbirth are the primary cause of decease (1 out of 7 girls) among under 19 years in third world countries [17].

On the other hand, advanced maternal age (> 30 years) at first birth is linked with an higher risk of miscarriage, chromosomal anomalies, multiple pregnancies, hypertension, diabetes mellitus, preterm birth, low birth weight, breast cancer and maternal mortality [16, 18, 19]. Those who gave first birth at 30 years and above group were 33% at higher risk of mortality compared to those who gave first birth in the age group 20–24 years in Ohsaki Japan [16]. The impacts of urbanization and modernization postponed the age at first birth in the later age in the developed world [9, 20–22].

Worldwide 20% of women give birth by the age of 18, in the poorest regions of the world, this rises to beyond a third (35%) in Kenya [14]. The median age at first birth in East Asia and Pacific was 20.2 years in Martial Island in 2007 and 23.4 years in Samoa in 2009 [8] in Bangladesh the mean age at first birth is 17.92 years [23], in Ghana 19.91 years in 2008 [24]. The median age at first birth in Nigeria was 20 years in 2013 [25]. In Ethiopia, more than a third (34%) of women age 20–49 give birth by the age of 18 and 54% by their age of 20 [22].The mean age of women at first birth was 18.47 years in Degua Tembien District, Tigray, Ethiopia [26].

There are numerous factors for this high prevalence of poor maternal and child health in third world nations. This includes deep-seated socio-cultural and spiritual practices, illiteracy and reduced income [13, 15, 16, 25, 27, 28]. Non-Muslim women have first births latter than women [23, 25].

Socio-demographic factors that were recognized as a predictor of age at first birth in various literatures include early age at first sexual intercourse [9, 20, 24], high Spousal age gap [5, 23, 29]. Some of the studies revealed that age at first birth at a lower age is higher in a woman of having wide Spousal age difference [5, 23, 29]. The younger age at first marriage is one of the most consistent findings across the studies as a risk factor for early age at first birth [5, 6, 9, 14, 17, 21, 23–25, 28, 30–35].

The Socio-economic factors were identified as a predictor of age at first birth as consistent manner. As many studies showed that the probability of early age at first birth is higher in women with no or lower level education compared to women at a higher educational level [1, 5, 6, 9, 14, 17, 20, 21, 23–25, 29, 32–34, 36, 37]. Most of the studies revealed that having poor and middle wealth index status was identified as the risk of age at first birth in early age.

Childbirth is being delayed to a later age with the mean age at first birth 26.3 years in the United States America (USA) [38] to 30 years in Britain [3]. The majority works of the literature revealed that the use of any contraception delay age at first birth [5, 8, 9, 23–25, 30, 39–41].

For most populations having first marriage at a lower age tend to have early childbearing and high fertility [6, 25]. On top of that in modern times many children are born before marriage with numerous health risks, like abortion and HIV [42]. The prevalence of premarital conceptions is 1% in Tanzania [43] and 1.2% in Ethiopia [44].

Even though the timing of first birth measured by the age at first birth has a huge effect on maternal and child survival, both individual and cumulative levels of fertility, as well as extensive implications on, women’s roles and social changes in general, studies conducted in Ethiopia on this topic are scarce. Furthermore, those limited studies conducted on teenage pregnancy [35]and age at first birth after marriage [34] did not account births outside marriage and first birth beyond adolescent period. Knowledge on the timing and factors of first birth in the country will help to design proper strategies to improve maternal and child health. Thus, by considering the above limitations this study is designed to estimate time to first birth and identify predictors among all reproductive-age women regardless of their marital status in Ethiopia with taking into account the correlated nature of the data. So, the study will serve for the next researchers and program planners to improve both maternal and child health with consideration of contextual/cluster effects.

## Methods

### Study design

Community based Cross sectional survey was conducted from January 18, 2016 to June 27, 2016 among reproductive-age women in Ethiopia [44].

### Study area and period

The study was conducted in Ethiopia one of the Sub-Saharan African country where the maternal mortality ratio 412 per 100,000 live births, skilled delivery coverage 28%,the median age at first marriage 17.1 years and the median age at first sexual intercourse 16.6 years, the contraceptive prevalence among married 36%, sexually active unmarried women 58% [44]. The estimated population in 2016 was 102 million with a fertility rate of 4.46 and the second largest population in Africa. The majority (78%) of women lived in rural [44]. The study was conducted from January 18 to June 27, 2016.

### Study participants

The study included all reproductive age-women (15–49 years) found in the selected clusters at least one night before data collection period January 18, 2016 to June 27, 2016. Taking reproductive age-women (15–49 years) of Ethiopian in place of source population, reproductive age women living in selected clusters as study population and reproductive age-women (15–49 years) found in 2016, Ethiopian demographic health survey (EDHS) enumeration areas at least one night before data collection as per Sample population [44]. Women declared infecund were excluded.

### Operational definitions

*Access to media* Respondents were asked how often they read a newspaper, listened to the radio, or watched television. Those who had exposure to one of them at least once a week are considered being regularly exposed to media [44, 45].

*Time to first birth* refers to the age of a mother in years when she gave birth to the first child after puberty [1, 2, 38].

*Censored* Those women who did not gave birth until the 2016 EDHS data collection end date.

*Event/Uncensored* mothers who gave first birth until 2016 EDHS data collection end date.

*Declared infecund* married or in union women for 5 + years, had no children in the past 5 years and never used contraception [45].

*Time to event/waiting time* it is the time in years from puberty to age at first birth.

*Beginning time* women at puberty (10 years from her birth date).

### Sampling technique and sample size determination

The 2016 EDHS sample was selected using stratified two-stage cluster sampling design and census enumeration areas (EAs) were the sampling units for the first stage and the detail published [46].

A total of 18,008 households were selected for the sample, of which 17,067 were occupied. Of the occupied households, 16,650 were successfully interviewed, yielding a response rate of 98%. In the interviewed households, 16,583 eligible women were identified for individual interviews. Interviews were completed with 15,683 women, yielding a response rate of 95% [44].

**Fig. 1:**
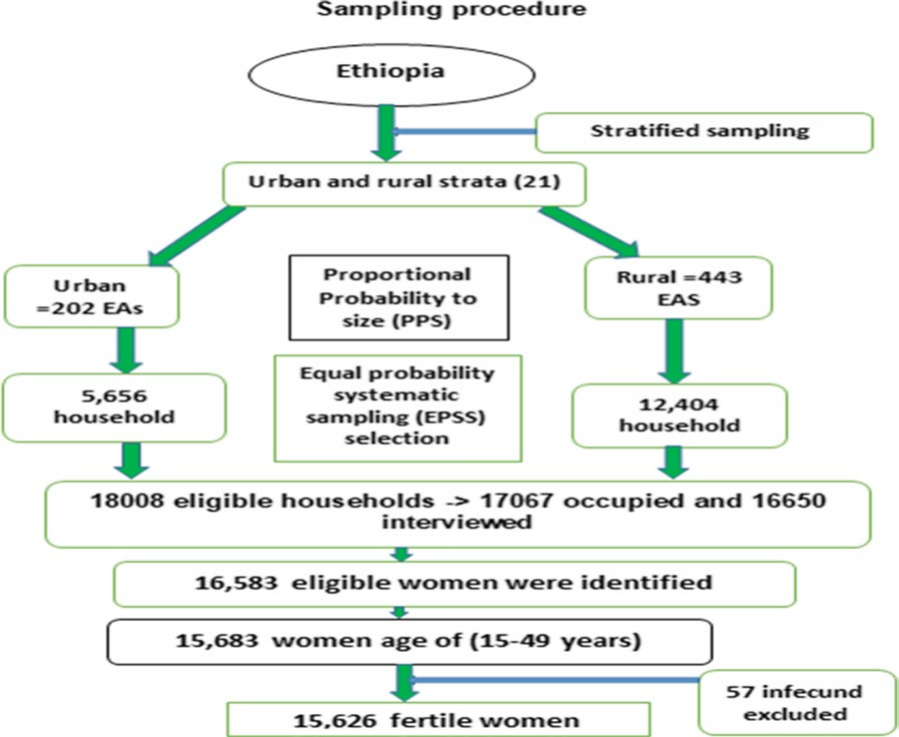
Sampling procedure of time to first birth and its predictors among reproductive age women in Ethiopia, 2016 EDHS

After the exclusion of primary infertile (57 women) from the data, the effective sample size became 15,626 (Fig. 1).

### Dependent and independent variable

The dependent variable in the current study is time to first birth in years when a woman gave her first childbirth until data collection period. The independent variables included, socio-demographic and reproductive health related factors (Age at first sexual intercourse, age at first marriage, Ever married, Spousal age difference); socio-economic and information related factors (respondent’s education, respondent’s occupation, Husband’s education, Husband occupation, Wealth index and Mass media exposure); Community level factors (region and residence) and Use of contraception as an immediate factor [46].

### Data source

For this study secondary data from the 2016 EDHS was used. The data set downloaded from the website https://dhsprogram.com after approval letter for use had obtained from the measure DHS. Variables were extracted from the EDHS 2016 individual women’s data set using a data extraction tool.

### Measurement of variables

Dependent variable, time to first birth measured in years was taken from age at first birth for mothers at least gave their first birth and the current age of respondent for event censored women. For the purpose of analysis those women gave birth event coded 1 (success) and those who did not give birth 0 (censored).

Independent variables age at first sexual intercourse and age at first marriage classified in to three categories; less than 15, 15–17 and 18 and above years, the highest age category taken as reference. Ever married coded as married and not married. Spousal age difference categorized as less than 5 years and 5 and above years. Respondent’s and husband education categorized into (no education, primary, secondary and higher education) and no education taken as reference. Respondent’s and husband occupation coded as not working, agriculture and non-agriculture with non-agriculture reference. Wealth index was classified as (poorest, poor, middle, richer and richest) by taking poorest as comparison group. Mass media exposure (yes/no), and use of contraception (yes/no). The regions were classified into six categories because there socio-cultural and economic similarities and geographical relations of the regions. These are northern regions (Amhara and Tigray), Oromia, Southern Nations, Nationalities and Peoples (SNNP), eastern pastoralist referring to the pastoralist dominant Afar and Somali regions, western region semi pastoralist representing Gambella and Benishangul-Gumuz, and most urban regions representing (Addis Ababa and Dire Dawa city administrations and Harari),while residence classified as urban and rural [46].

### Data quality control

After all, questionnaires were finalized in English; they were translated into local languages (Amarigna, Tigrigna, and Oromiffa) and pretested at Bisheftu. Computer-assisted personal interview data collection system was carried out to collect data by trained EDHS data collectors and mobile version CSPro software was used for entering and capturing the data [44].

For this study the same source population used for both those who gave birth or not to make comparable. The data collectors and study participants were blind to the study hypothesis since the analysis considered later. Data extraction checklist was prepared and data extracted using Stata version 14.0.

### Data analysis

After the data were extracted, cleaned and weighted descriptive measures such as median, percentiles, graphs and frequency tables were used to characterize the study population. We estimated time to first birth using the Kaplan–Meier (K–M) method and compared across categorical predictor variables using log rank test. Schoenfeld residual test was applied to check the proportional hazard assumption.

Since our data were correlated at cluster level, shared frailty model were modeled by taking enumeration areas/clusters as a random effect for predictors of time to first birth among reproductive-age women in Ethiopia assuming time to first birth to be constant in the same clusters. The efficient model was selected by the smallest AIC value. Model adequacy was checked using Akaike Information Criteria (AIC), Cox-Snell residuals and R^2^ type statistic.

Stratified analysis and chi-square test were applied for interaction terms. Finally adjusted hazard ratio (AHR) and adjusted time ratio (ATR) as a measure of effect size reported at 5% significant level and *p* value < 0.05.

Stata 14.0/SE software for data extraction, cleaning and analysis was used.

### Measures of dependence in shared frailty modeling

The associations within group members are measured by Kendall’s, which is given by

$$\tau = \frac{\theta }{{\uptheta + 2}}$$

where τ ɛ [0, 1].

For Gamma frailty distribution (θ > 0)

$$\tau = \frac{1}{2} - \frac{1}{\uptheta } + \frac{2}{{\uptheta \frac{2}{{}}}}e ^{{\left( {\frac{2}{\uptheta }} \right)}} \mathop \smallint \limits_{{2/\uptheta }}^{\infty } u^{ - } e^{{\left( { - u} \right)}} du < \frac{1}{2}$$

where τ ɛ (0, 1/2).

For Inverse Gaussian frailty distribution (θ > 0).

The median hazard ratio (MHR) was used to compare between high and low risk clusters of time to first childbirth.

MHR = $e^{{\left( {\sqrt {2\theta } *\Phi^{ - 1} *\left( \frac{3}{4} \right)} \right)}}$ where θ = variance of frailty, $\Phi$^−1^ = inverse normal distributions.

## Ethical consideration

The written approval letter was obtained from the DHS International Program to use the data for this analysis which authorized for the data-sets. Before data collection EDHS data collection materials were approved for compliance of the requirements of 45 CFR 46, “Protection of Human Subjects” by Institutional Review Board (IBR) with ICF Project Number: 132989.0.000.ET.DHS.01. Complete information regarding the ethical issue was available in the EDHS-2016 report [44].

## Results

### Baseline characteristics of study participants

A total of 15,626 (weighted = 15,635) women were included in the study. From the respondents one fifth (21.62) were adolescents. Of all, the majority (77.88%) of them were rural in residence. Of married women, 67.37% had more than 5 years of a spousal age difference. Around two-thirds (63%) of women married their first husband before their age of 18 years. Moreover, 26.24% of them married before the age of 15 years (Table 1).

**Table 1:** Socio-demographic and reproductive health-related factors among reproductive age women in Ethiopia, EDHS 2016

Variables	Categories	Weighted frequency	Weighted percentage (%)
Age distribution (N = 156,356)	< 20 years	3380	21.62
	20–29 years	5716	36.56
	> 30 years	6539	41.82
Residence	Urban	3447	22.12
	Rural	12,175	77.88
Regions	Most urban	1042	6.74
	Northern	4827	30.87
	Oromia	5682	36.34
	SNNp	3282	21.00
	Eastern-pasto	585	3.74
	Western-Pasto	204	1.30
Age at first intercourse (n = 15,635)	< 15 years	3027	19.36
	15–17 years	5062	32.38
	> 18 years	7546	48.26
Age at first marriage (n = 11,600)	< 15 years	3044	26.24
	15–17 years	4256	36.69
	> 18 years	4300	37.07
Spousal age difference	< 5 years	3326	32.63
	> 5 years	6867	67.37
Contraceptive	Ever not use	8895	56.95
(n = 15,635) religion	Ever use	6727	43.05
	orthodox	6,762	43.25
	Muslim	4,881	31.22
	protestant	3,662	23.42
	Others^a^	330	2.11

^a^Others = catholic, traditional and other

Regarding socio-economic and information related characteristics of the respondents, 47.77% of them had no education and 49.91% have no formal occupation. More than 34% of the study participants had wealth index status below the middle level and only a quarter (26.37%) had regular media exposure (Table 2).

**Table 2:** Socio-economic and information related characteristics among reproductive age women in Ethiopia EDHS, 2016

Predictors	Categories	Weighted frequency	Weighted percentage (%)
Education	No education	7469	47.77
	Primary	5475	35.04
	Secondary	1802	11.61
	Higher	868	5.57
Occupation	Not working	7799	49.91
	Agriculture employee	3248	20.77
	None agriculture employee	4576	29.32
Husband education	No education	4750	46.60
	Primary	3765	36.94
	Secondary	971	9.53
	Higher	707	6.94
Husband occupation	Not working	802	7.87
	Agriculture employ	6323	61.93
	Non-agri-employe	3078	30.20
Media exposure	Yes	4115	26.37
	No	11,508	73.63
Wealth index	Poorest	2630	16.82
	Poorer	2803	17.93
	Middle	2968	18.98
	Richer	3092	19.77
	Richest	4143	26.49

### Time to first birth among study participants

**Fig. 2:**
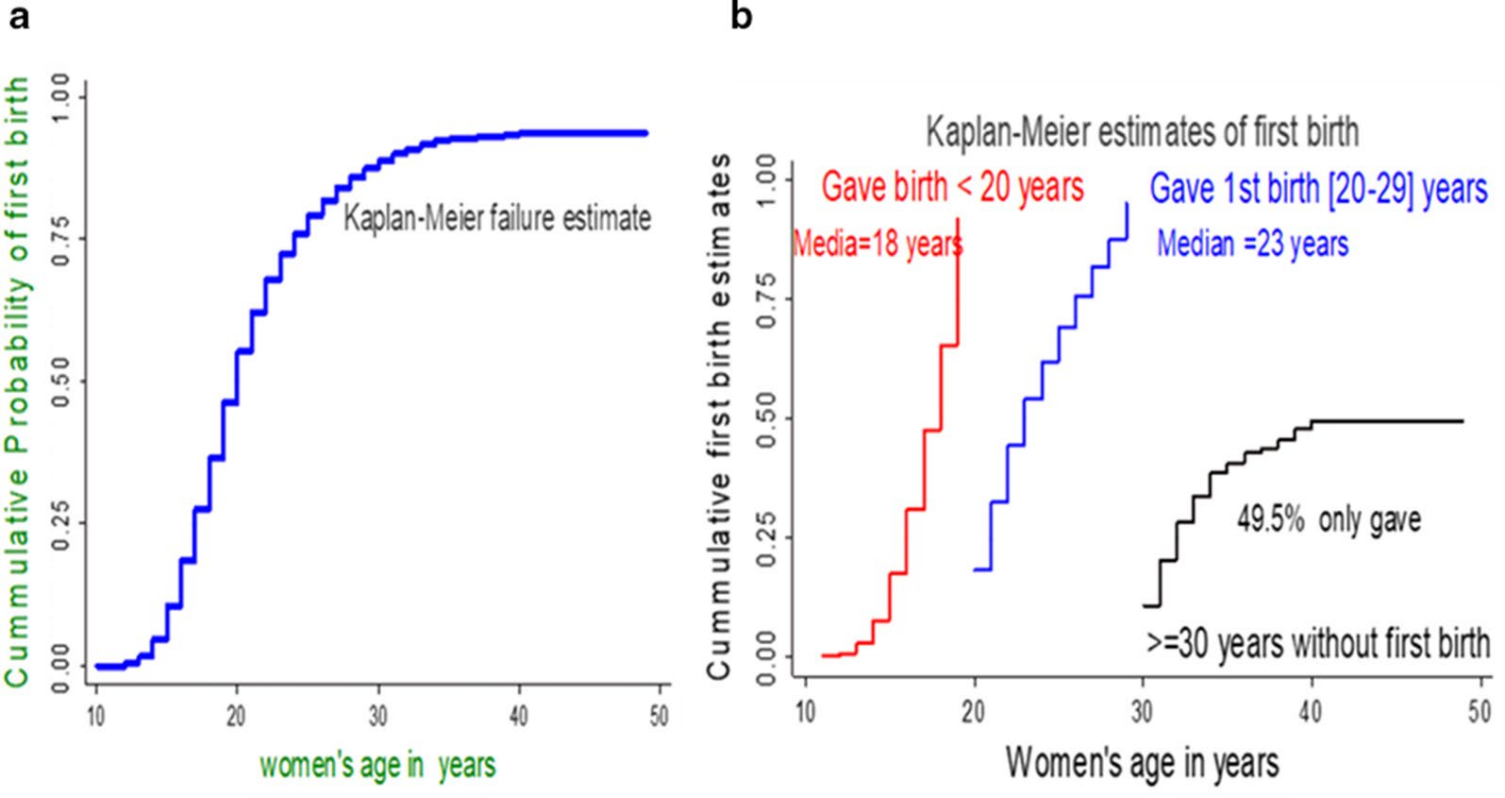
Kaplan–Meier failure estimates of time to first birth among reproductive-age women in Ethiopia, 2016 EDHS. **a** Overall Kaplan–Meier failure estimate. **b** Early, optimal and advanced age at first birth estimates among respondents

Over all 10,274 (67.7%) women had given at least first birth. The total follow-up period for all 15,626 women was 146,290 person-years of observation. The median, minimum and maximum follow-up period was 8 years 1 year and 39 years after the age of puberty (10 years from her birth date), respectively. The overall median time to first birth was 20 years (IQR = 16–24). Among women had first birth below 20 years the median age was 18 years (IQR = 15–19), from those women giving first birth in the age bracket of 20–29 years median age was 23 years (IQR = 20–26) and of women celebrate their 30 years before giving birth (n = 533) only 49.5% able to give birth (Fig. 2).

The median time to first birth was at lower age for those women enter into sexual intercourse at lower age (< 15 years) 15 years (IQR = 13–17), too early married women (below 15 years) 16 years (IQR = 14–17) and women had no education 18 years (IQR = 15–21) years (Table 3). The median age was relatively higher among women those had higher education level 27 years (IQR = 21– –).

**Table 3:** Kaplan–Meier failure estimate and log rank test comparison of time to first birth among reproductive age women in Ethiopia, 2016 EDHS

Characteristics	N (%)	Ever given birth	Median (IQR) years	Log-rank	*p* value
Region					
Northern	4827 (30.87)	3224 (66.8)	19 [16–24]	570.2	< 0.001
Oromia	5682 (36.34)	4130 (72.7)	19 [16–22]		
SNNPR	3282 (21.00)	2192 (66.8)	20 [16–24]		
Most-urban	1042 (6.74)	474 (45.5)	26 [19–34]		
Eastern-pasto	585 (3.74)	424 (72.4)	19 [16–22]		
Western-semi Pasto	204 (1.30)	144 (70.7)	19 [16–22]		
Residence					
Urban	3447 (22.12)	1769 (51.3)	23 [18–30]	916.7	< 0.001
Rural	12,175 (77.88)	8818 (72.4)	19 [16–22]		
Education					
No education	7469 (47.77)	6785 (90.9)	18 [15–21]	2545.8	< 0.001
Primary	5475 (35.04)	2865 (52.3)	20 [17–24]		
Secondary	1802 (11.61)	593 (32.8)	26 [20–37]		
Higher	868 (5.57)	344 (39.6)	27 [21–49]		
Occupation					
Not working	7799 (49.91)	5243 (67.2)	19 [16–23]	461.0	< 0.001
Agriculture	3248 (20.77)	2522 (77.6)	19 [15–21]		
Non agricul-	4576 (29.32)	2822 (61.8)	21 [17–27]		
Wealth index					
Poorest	2630 (16.82)	2060 (78.3)	19 [16–22]	903.9	< 0.001
Poorer	2803 (17.93)	2122 (75.7)	19 [16–21]		
Middle	2968 (18.98)	2128 (71.7)	19 [16–22]		
Richer	3090 (19.76)	2089 (67.6)	19 [16–23]		
Richest	4131 (26.49)	2190 (53.0)	22 [17–30]		
Contraceptive					
Ever not use	8895 (56.95)	4617 (51.9)	21 [17–26]	844.2	< 0001
Ever use	6727 (43.05)	5970 (88.7)	19 [16–22]		
Media exposure					
No	11,508 (73.63)	8342 (72.5)	19 [16–22]	576.6	< 0.001
Yes	4115 (26.37)	2245 (54.6)	22 [17–28]		
Ever married					
No	4022 (25.81)	93 (2.3)	–	3974.7	< 0.001
Yes	11,600 (74.19)	10,494 (90.5)	19 [15–21]		
Spousal age gap					
< 5 years	3326 (32.63)	2984 (89.7)	19 [16–22]	129.2	< 0.001
≥ 5 years	6,867 (67.37)	6366 (92.7)	18 [15–21]		
Age at first marriage					
Below age 15	3044 (26.24)	2915 (95.8)	16 [14–17]	5582.3	< 0.001
15–17	4256 (36.69)	3890 (91.4)	18 [16–19]		
18 and above	4300 (37.07)	3689 (85.8)	22 [19–25]		
Age at first sex					
< 15 years	3027 (19.36)	2860 (94.47)	15 [13–18]		
15–17 years	5062 (32.38)	4576 (90.4)	18 [16–20]		
≥ 18 years	7546 (48.26)	3151 (41.75)	22 [19–26]		
Husband-education					
No education	4750 (46.60)	4516 (95.1)	18 [15–21]	396.1	< 0.001
Primary	3765 (36.94)	3454 (91.7)	18 [15–21]		
Secondary	971 (9.53)	808 (83.2)	20 [16–23]		
Higher	707 (6.94)	573 (81.1)	21 [17–25]		
Husband occupation					
Not working	802 (7.87)	740 (92.3)	18	256.1	0.001
Agriculture employee	6323 (61.93)	5930 (93.9)	18		
Nonagriculture	3078 (30.20)	2681 (87.1)	19		
Religion					
Orthodox	6762 (43.25)	4371 (64.6)	20	178.6	< 0.001
Muslim	4881 (31.22)	3593 (73.6)	19		
Protestant	3662 (23.42)	2367 (64.7)	20		
Others	330 (2.11)	255 (77.1)	19		
Total	15,635	10,635 (67.7)	20 [16, 24]		

*N* weighted value, *IQR *interquartile range

### Predictors of time to first birth among reproductive-age women in Ethiopia, 2016 EDHS

**Fig. 3:**
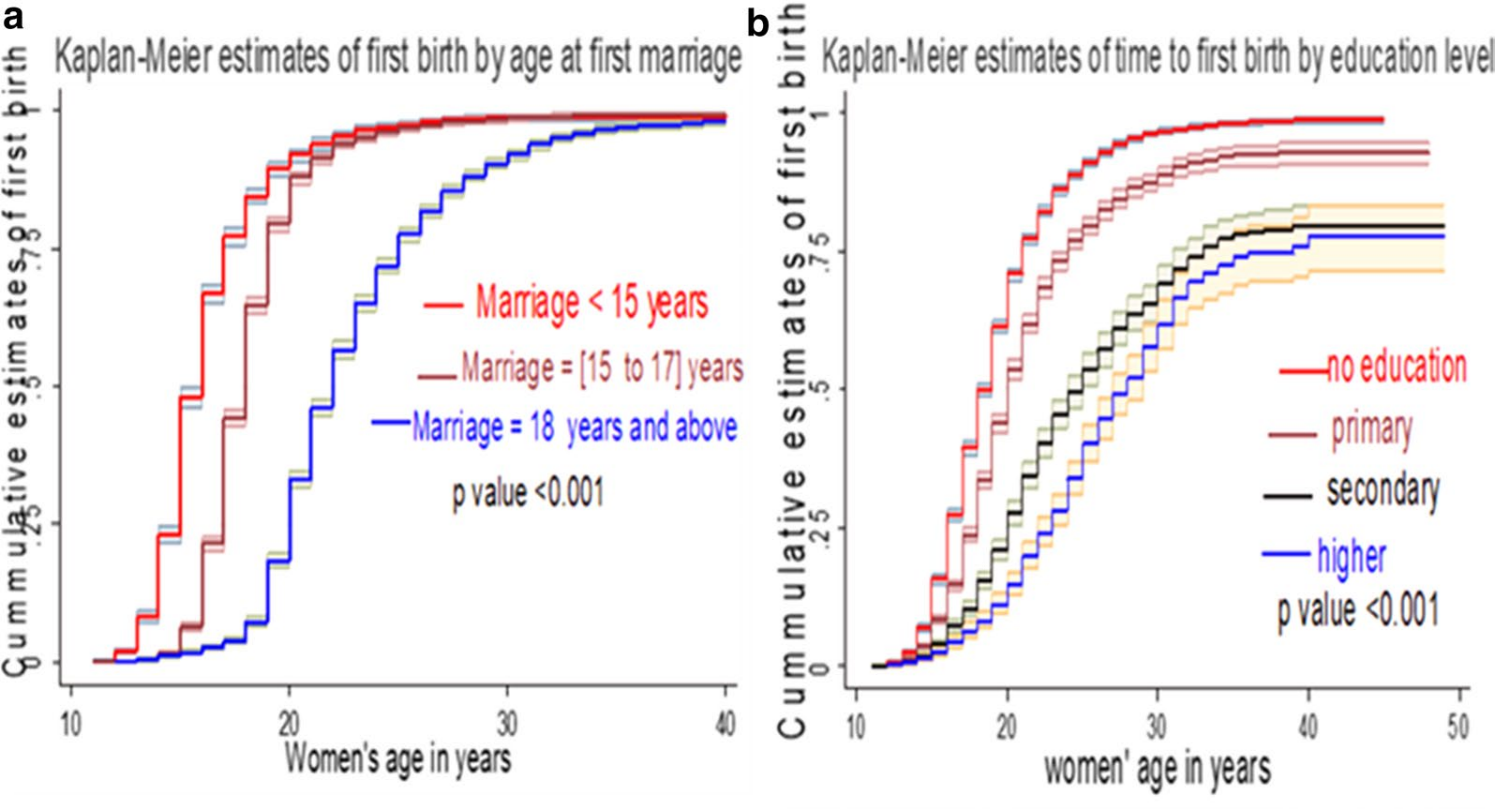
Kaplan–Meier failure estimates difference and log-rank equality of survival tests of time to first birth among reproductive-age women in Ethiopia, 2016, EDHS*.* **a** Kaplan–Meier estimate of time to first birth by age at first marriage. **b** Kaplan–Meier estimate of time to first birth by education level

Differences in all predictors at baseline were determined using the Kaplan Meier failure function and the log-rank (χ^2^) test. The Kaplan Meier failure function with 95% confidence interval was constructed for age at first marriage and women education level (Fig. 3).

In general, the pattern of the failure function lying below right side to other categories indicated that the group defined by the lower curve had a better survival before giving first birth than the group defined by the above curves. Therefore women married at the age of 18 and above years and women with secondary and higher education level able to delay their first birth in the later age than married below 18 years and below secondary education level respectively as Kaplan Meier failure graph and a log-rank test at (*p* value < 0.001) showed. The log-rank test showed that all predictor variables had a significant survival difference at *p* value < 0.001 (Table 3).

### Parsimonious model selection

#### Cox proportional hazard model

All fourteen predictor variables that were significant at 0.2 *p* value in Bivariable analysis were entered into the multivariable Cox model and ever married reduced from the model due to collinearity effect. Then the Schoenfeld test for proportional hazard assumption of the time to first birth data was evaluated. The proportional hazard assumption violated in both global test and rank test due to significant correlation of time to first birth (Table 4), as a result, the Cox model was excluded for this data.

**Table 4:** Schoenfeld residual test for proportionality assumption of the Cox model

Predictors	Rho	Chi^2^	*df*	Prob > chi^2^
Geo-regions	0.056	91.18	1	< 0.001
Residence	− 0.078	174.81	1	< 0.001
Religion	− 0.061	82.37	1	< 0.001
Education level	0.071	107.88	1	< 0.001
Occupation	− 0.055	80.36	1	< 0.001
Wealth index	− 0.095	235.12	1	< 0.001
Spousal age difference	− 0.108	231.14	1	< 0.001
Contraceptive use	0.193	1009.79	1	< 0.001
Media exposure	0.030	20.33	1	< 0.001
Age first marriage	0.220	1589.96	1	< 0.001
Age first sex	0.138	463.55	1	< 0.001
Husband education	− 0.066	118.00	1	< 0.001
Husband occupation	0.019	13.02	1	< 0.001
Global test		6191.37	13	< 0.001

< 0.001 means significant at 5% significance level; proportionality assumption is violated

**Fig. 4:**
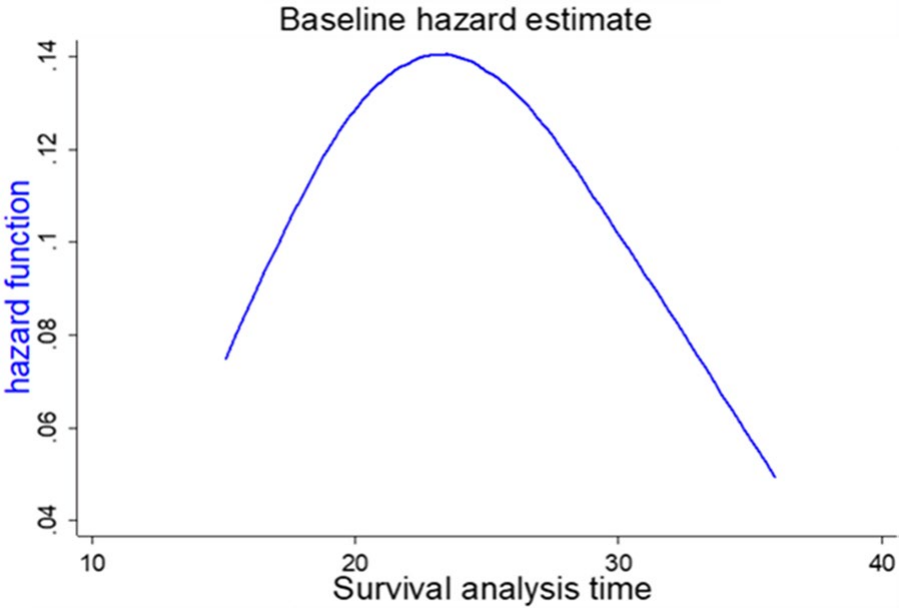
Base line hazard estimate of time to first birth among reproductive-age women in Ethiopia, 2016, EDHS

Stratified Cox model was also inappropriate for this data because there is no predictor variable that fulfills proportional hazard assumption to be in the model. Another alternative time-varying Cox model also faces the challenge of choosing the appropriate function of survival time to include in the model. However, in the case of our data, the time distribution is somewhat follow unimodal distribution (Fig. 4). So, those parametric models were considered.

### Appropriate parametric survival model selection

#### Parametric shared frailty model

A likelihood ratio test for a variance of frailty theta = 0 yields a highly statistically significant *p* value of < 0.001 for all baseline hazard function with both inverse Gaussian and gamma shared frailty distributions, suggesting that the frailty component contributes to the model and that there is a within-cluster correlation. The inverse Weibull gamma shared frailty model is preferred model for the give data due to its lowest AIC (Table 5).

**Table 5:** Parametric shared frailty model comparison on time to first birth data of reproductive age women in Ethiopia, 2016 EDHS

Model	Log-likelihood	DF	AIC	Variance of θ	LR test of θ = 0
Lognormal gamma	4511.66	34	− 8955.31	0.18	< 0.001
Lognormal inverse Gaussian	4511.62	34	− 8955.24	0.25	< 0.001
Log logistic gamma	4881.54	34	− 9695.08	0.23	< 0.001
Log logistic inverse Gaussian	4936.02	34	− 9804.03	0.87	< 0.001
Inverse Weibull gamma	5708.19	34	− 11,348.37	0.028	< 0.001^a^
Inverse Weibull inverse Gaussian	5707.78	34	− 11,347.55	0.028	< 0.001

^a^Preferred model

### Multivariable analysis of inverse Weibull gamma shared frailty model for time to first birth and its predictors among reproductive-age women in Ethiopia, EDHS, 2016

In the inverse Weibull gamma shared frailty model, the null model, only with the cluster effect and the full model, with predictor factors were compared to visualize reduction of frailty variance on the addition of predictor variables which revealed that in the full model variance theta reduced from null model 0.05–0.028 and 0.025. In this model predictor variables geographical regions, women education level, contraceptive use, spousal age difference, age at first marriage, age at first sexual intercourse, religion and age at first sexual intercourse interaction with age at first marriage, were significant predictor variables at 95% confidence level.

Having the same frailty or cluster effect living in Oromia increased the hazard of early childbirth by 18% (AHR = 1.18, 95%, CI 1.06–1.30); living in SNNP increased the hazard of early childbirth by 19% (AHR = 1.19, 95, CI 1.06–1.30); living in Eastern pastoralist region increased the hazard of early childbirth by 16% (AHR = 1.16, 95%, CI 1.05–1.28) and living in western semi pastoralist regions increased the hazard of early childbirth by 37% (AHR = 1.37, 95%, CI 1.24–1.52) than living in most urban regions controlling for other factors.

With the same level of frailty and adjusting for the other factors women having secondary and higher education level have 14% (AHR = 0.86, 95% CI 0.78–0.96) and 25% (AHR = 0.75,95% CI 0.65–0.85) hazard reduction of first birth at early age compared to women with no education level respectively.

Women with richer wealth index were had 10% higher hazard of first birth at an early age compared to those women with poorest wealth index keeping other factors constant and in the same frailty level (AHR = 1.10, 95%, CI 1.01–1.19).

Women living in the same cluster and adjusted for other factors women ever using any methods of contraceptive to delay first birth reduces the hazard of first birth at an early age by 0.91 times compared to ever non-users (AHR = 0.91, 95% CI 0.86–0.97).

Adjusting for other factors and women in the same frailty having spousal age difference greater than 5 years had 11% higher hazard of first birth at an early age compared to women having spousal age difference less than 5 years (AHR = 1.11, 95% CI 1.05–1.16).

At the same level of susceptibility and holding constant other factors women who were married 15–17 years had 2.33 (AHR = 2.33, 95%, CI 2.08–2.63) times higher hazard of first birth at early age compared to women those who were married 18 years and above respectively.

The hazard of first birth at an early age was increased by (AHR = 23.81, 95%, CI 22.22–25.64) times in married stratum and reduced by (AHR = 0.063, 95%, CI 0.035–0.11) time in not married stratum among women who were started sexual intercourse earlier than 15 years than those women started sexual intercourse at the age of 18 years and later with marriage in the same level frailty level and making constant other factors. The hazard of early childbirth was higher among women who were stated intercourse 15–17 years in marriage by (AHR = 5.56, 95%, CI 5.26–5.88) and it was reduced by (AHR = 0.033: 95%, CI 0.022–0.048) in those started intercourse before marriage than those who were started sexual intercourse in marriage at the age of 18 years and later.

Prior to adjusting for predictors the median increase in the hazard of early childbirth when comparing a woman at a cluster with higher risk of early childbirth to a woman at a cluster with lower risk early childbirth was 24% (MHR = 1.24, 95% CI 1.21–1.27) higher. After accounting for predictors and interaction term the median increase in the hazard of early childbirth when comparing a woman at a cluster with higher risk of early childbirth to a woman at a cluster with lower risk of early childbirth was 16% (MHR = 1.16, 95% CI [1.13–1.20]) (Table 6).

**Table 6:** Bivariable and multivariable inverse-Weibull gamma shared frailty model on predictors of age at first birth among reproductive-age women in Ethiopia, EDHS, 2016

Variable	Null model	First birth status			Full model
Log-likelihood	− 1036.84			5907.45	
Effect size		Gave	Not gave	CHR	AHR
Region					
Most urban		474	568	1	1
Northern		3224	1603	1.08 (1.01–1.18)**	1.08 (0.97–1.19)
Oromia		4130	1552	1.07 (0.98–1.18)*	1.18 (1.06–1.30)***
SNNP		2192	1090	0.98 (0.90–1.18)	1.19 (1.06–1.33)***
Eastern		424	161	1.00 (0.92–1.09)	1.16 (105–1.28)***
Western		144	60	1.12 (1.03–1.22)**	1.37 (1.24–1.52)***
Residence					
Urban		1769	1678	1	1
Rural		8818	3357	0.96 (0.91–1.02)	1.09 (0.98–1.19)
Education					
No education		6785	684	1	1
Primary		2865	2610	1.72 (1.64–1.82)***	1.12 (1.05–1.19)
Secondary		593	1209	0.83 (0.76–0.87)***	0.86 (0.78–0.96)***
Higher		344	524	0.72 (0.65-.80)***	0.75 (0.65–0.85)***
Occupation					
Not working		5243	2556	1.18 (1.13–1.24)***	0.98 (0.92–1.04)
Agriculture		2522	726	0.98 (0.93–1.04)	0.94 (0.88–1.02)
Nonagriculture		2822	1754	1	1
Wealth index					
Poorest		2060	570	1	1
Poorer		2122	681	1.03 (0.96–1.10)	0.98 (0.91–1.05)
Middle		2128	840	1.12 (1.05–1.21)***	1.00 (0.93–1.09)
Richer		2089	1001	1.12 (1.05–1.21)***	1.10 (1.01–1.19)*
Richest		2190	1941	1.12 (1.05–1.21)***	1.08 (0.97–1.15)
Spousal age diffe					
< 5 years		2984	342	1	1
≥ 5 years		6366	501	1.26 (1.21–1.33)***	1.11 (1.05–1.16)***
Contraceptive					
Ever not use		4617	4278	1	1
Ever use		5970	757	0.50 (0.48–0.52)***	0.91 (0.86–0.97)***
Media exposure					
No		8342	3166	1	1
Yes		2245	1870	1.10 (1.04–1.15)***	1.01 (0.94–1.08)
Age at first marriage					
< 15 years		2915	129	9.10 (8.83–9.86)***	1.26 (0.87–1.82)
15–17 years		3890	366	3.47 (3.30–3.64)***	2.33 (2.08–2.63)***
≥ 18 years		3689	611	1	1
Age at first sex					
< 15 years		2860	167	1.89 (1.78–1.99)***	27.78 (23.26–32.26)***
15—17 years		4576	486	0.68 (0.65–0.71)***	2.60 (2.07–2.63)***
≥ 18 years		3151	4395	1	1
Husband education					
No Edu-		4516	234	1	1
Primary		3454	311	0.99 (0.94–104)	1.03 (0.97–1.09)
Secondary		808	163	0.88 (0.81–0.95)***	1.05 (0.96–1.15)
Higher		573	134	0.68 (0.63–0.74)***	1.03 (0.93–1.14)
Husband occupation					
Not working		740	62	1.14 (1.05–1.23)***	0.98 (0.91–1.o6)
Agriculture		5930	393	1.13 (1.08–1.19)***	0.96 (0.91–1.02)
Non agriculture		2681	397	1	1
Religion					
Orthodox		4371	2391	1	1
Muslim		3593	1288	1.02 (0.97–108)	1.10 (1.03–1.19)***
protestant		2367	1295	0.99 (0.92–1.05)	1.01 (0.92–1.10)
Others		255	75	0.90 (0.77–1.06)	1.03 (0.87–1.22)
> 18intercourse and marriage					1
< 15marriege < 15 intercourse					1.32 (0.62–2.89)
< 15 marriage 15-17intercourse					0.86 (0.62–1.23)
15–17 intercourse < 15marriage					4.55 (2.13–10.02)***
15–17 intercourse 15–17 marriage					2.63 (2.01–3.45)***
Theta	0.05 (0.04–0.06)				0.025 (0.016–0.039)***
MHR	1.24 (1.21–1.27)				1.16 (1.13–1.20)***
LR test of theta = 0					
Chibar2(1)	126.23				35.40
Prob-hibar2	< 0.001				< 0.001

*significant at 90 % Confidence level; **significant at 95% confidence level; ***significant at 99% confidence level

MHR = median hazard ratio, † from interaction model

The hazard of early childbirth was higher among Muslim religion followers by 10% than orthodox followers given that they were in the same cluster and control for other factors (AHR = 1.10, 95%, CI 1.03–1.19).

In addition to main effect the interaction term revealed that those married before the age of 15 and enter into sexual intercourse at the age of 15–17 years had an increased hazard of first birth at an early age by (AHR = 4.55, 95% CI 2.13–10.02) than who were enter into sexual intercourse with marriage at the age of 18 and later. The hazard of an early childbirth increased by (AHR = 2.63, 95% CI 2.01–3.45) times among women had sexual intercourse at marriage 15–17 years than who were married 18 years and later.

## Model adequacy

**Fig. 5:**
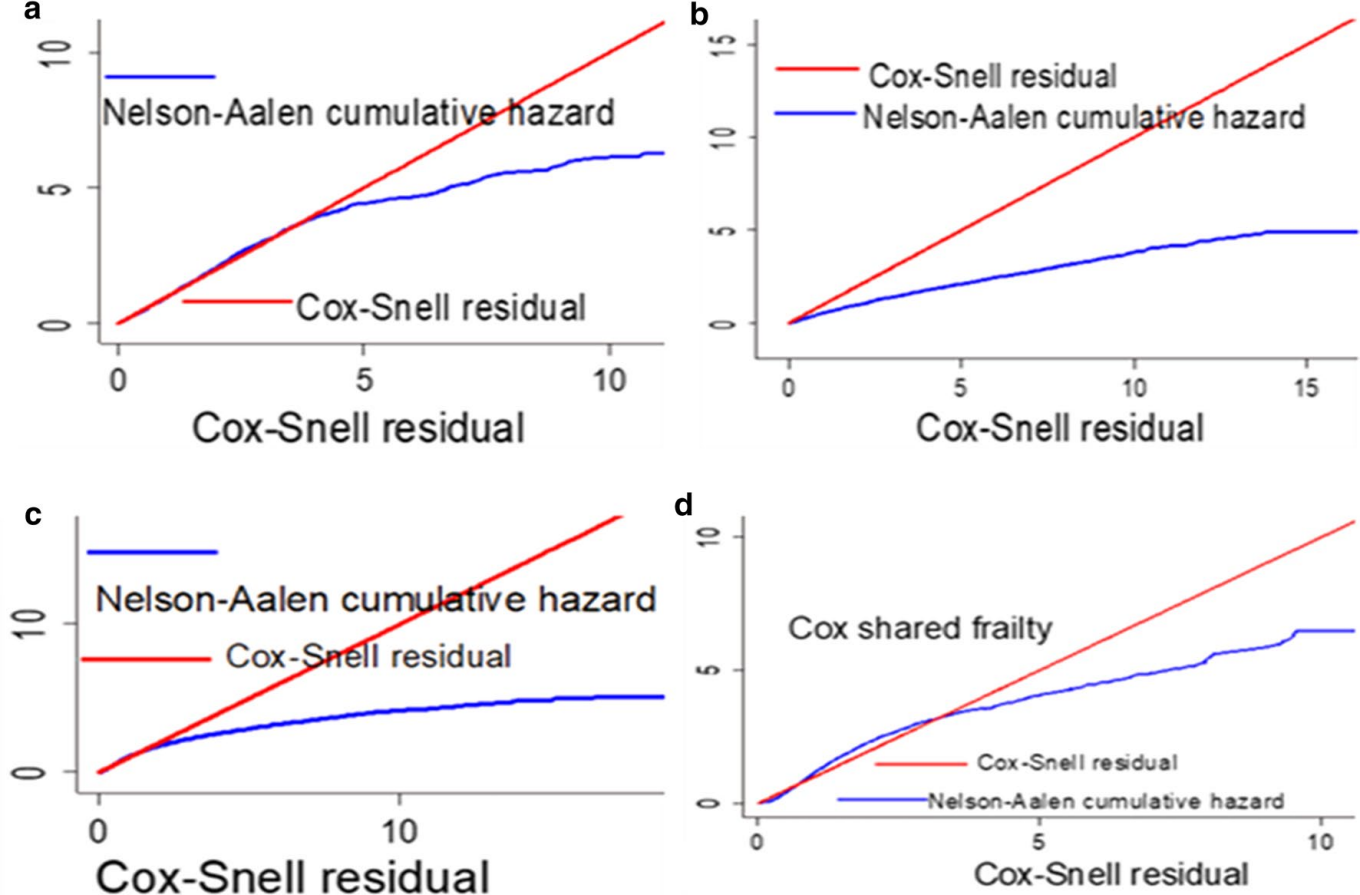
Cox–Snell residual and Nelson Aalen cumulative hazard plots of time to first birth in Ethiopia, 2016, EDHS. **a** Inverse-Weibull gamma shared frailty. **b** Log–logistic inverse Gaussian shared frailty. **c** Lognormal gamma shared frailty. **d** Cox gamma shared

The Cox-Snell residuals versus the Nelson-Aalen cumulative hazard function were obtained by fitting the cox gamma shared frailty, inverse Weibull gamma shared frailty, log-logistic inverse Gaussian frailty and lognormal gamma shared frailty models. The Nelson Aalen cumulative hazard function against the Cox-Snell residuals has a linear pattern making a straight line through the origin of the inverse Weibull gamma shared frailty model when compared to the rest models. This suggests that the inverse Weibull gamma shared frailty model provided the best fit for the time to first birth data analysis (Fig. 5).

## Discussion

This study was set out to examine the timing of first birth among reproductive-age women in Ethiopia and modeled factors affecting it using a parametric shared frailty analysis method. The study revealed that majority of women gave their first birth at an early age and age at first marriage, age at first sexual intercourse and women education were most significant factors.

In the current study, the median age at first birth found to be 20 years. This finding is in agreement with the finding of, Martial Island, Ghana, and Nigeria where the median age at first birth was 19.91 years in Ghana, 20 years in Nigeria to 20.2 years in Martial Island [8, 24, 33]. This might be due to the high prevalence of early marriage and sexual intercourse activities in these countries [47, 48]. Early marriage compromise women’s decision role in her reproductive health and resulted in early childbirth [49]. The other possible justification for this similarity might be due to the limited educational opportunity of girls in these countries as the majority of the population lived in the rural area [50], which forces them to get married at an early age, to get social and financial support [51, 52].

However, our result was far lower than the median ages (> 30 years) in most developed countries [3, 53]. This might be due to a higher opportunity for girls to stay in school for their adolescent age and a number of women going out to work for their economic independence [3] which help mothers to delay their first birth. It is also well justified with the awareness of women in developed countries about the consequences of early childbirth and having access to contraceptives to delay first birth in these countries [51]. Moreover, women in developed countries have the chance to exercise their reproductive rights and make a decision regarding their reproductive health issues [51].

On the other hand, our finding was higher than the finding in in Degua Tembien District, Tigray, Ethiopia [26] and in Bangladesh [23]. This variation could be due to difference in study design and small sample size in the former study and difference in study period which was in 2011 Bangladesh Demographic and Health Survey (BDHS). Moreover, in the latter case the variation might also be explained with difference in socio-cultural and religious affiliation, since 89% of the population Bangladesh was Muslims [54] whereas not more than 31% in Ethiopia [44]. Muslim women have their first births earlier than non-Muslim women [23, 25].

Women who were started sexual intercourse at an early age had higher hazards of having first birth at an early age than those who were started intercourse at a later age. This outcome is in concurrence with studies conducted in Ghana [24], Bangladeshi [20] and Swaziland [9]. This might be due to the exclusion of adolescents from education and sociocultural miss-conception regarding female reproductive health issues and poor legal backing of women in these developing countries. This argument is supported by the United Nation (UN) report stating that still, one-half (49.8%) of the female youth population had either no education or limited education in developing countries [55].

Age at first cohabitation was also another predictor of time to first birth, as women got married early, the hazard of early motherhood at an early age was increased. This is in agreement with studies conducted in Bangladesh [23],Nigeria [33] and other many studies conducted elsewhere [3, 4, 7, 14, 19, 20, 22, 26, 31, 32]. It is apparent that marriage increases the frequency of fertile sexual intercourses and as it happened in early, it leads motherhood at an early age [22]. This is consistent with previous evidence that shows marriage is not planned and desire for women in developing countries rather a requirement to get an economic guarantee and social respect by keeping virginity at marriage [52]. In most cases, young housewives are characterized by lower educational attainment, lack of adequate formation about the negative consequences of early childbearing, are economic dependent on their spouses and limited to no role in the decision-making process which fundamentally restricts their capability to delay their childbearing to older ages [49].

In addition to main effects the interactions terms between age at first sexual intercourse and age at first marriage revealed those in married stratum in early age were positively associated with early age childbirth and those in not married stratum were negatively associated with early childbirth. It might be due to that in Ethiopia marriage provides normative legalization for childbirth. So, even sexual intercourse happened in an early age unless supported with marriage probability of childbirth is minimal in Ethiopia.

Higher spousal age gap among women was found to be linked with higher early age maternity. This result was in agreement with reports in Nigeria [33] and Bangladesh [23]. Possible reasons might be that higher spousal age difference may cause imbalanced power relations in the family and low level of inter-spouse communication which fundamentally translate into women’s participation in the family decision-making process including the decision to use contraceptives [49, 52].

Women’s education and early motherhood were inversely associated in this study. This finding was corroborate with study findings in Degua Tembien District, Tigray, Northern Ethiopia [26], Bangladeshi [1] and results elsewhere [3, 4, 7, 14, 19, 21, 22, 26, 31–35, 41–43]. In particular, ensuring those adolescent girls to receive at least a secondary level of education is the optimal way of delaying childbirth [53, 58]. Possible explanations of the inverse association between educational attainment and motherhood at an early age could be due to that enrolling and retaining girls at least up to a secondary level of education probably reduce early marriage and sexual experience and increase awareness of reproductive health issues [50, 56]. In contrast, women at lower education level have lack of adequate knowledge about the high-risk period of becoming pregnant, are not fully aware of family planning methods and the costs of early childbearing on mothers and children health [57].

Regarding region of residence Oromia, SNNP, Eastern pastoralist and western semi-pastoralist regions significantly increased the hazard of first birth at an early age compared to most urban regions (Addis Ababa, Dire Dawa, and Harare), controlling for other factors and holding cluster effect the same. This finding was coherent with the study reported in Ethiopia [58] and Ghana [24].This might be due to fewer proportions of educated women, and access to contraceptive and reproductive health issues in rural regions [44]. Furthermore, women in rural regions might have less decision making role regarding their reproductive health and timing of first birth.

One contrasting finding in this study was the richer wealth index associated with an increased hazard of first birth at an early age compared to the poorest in the full model. The possible reason may be as 78% of women in Ethiopia are rural dwellers [44] and in most rural areas wealth is one precondition for marriage as “macha”, which is an amalgamation of wealth from families for the new couples. So those girls from richer families got married early in their lives and became mothers in adolescent age.

Ever use of contraceptive linked to the delay of the first birth than none user counterparts in this study. This result is in agreement with findings documented in Northeast Ethiopia [39], East Asia [8] and studies elsewhere [3, 7, 19, 31, 33, 37, 40]. It might be due to the fact that as far as the appropriate utilization of contraceptive, sexually active women may able to delay unintended pregnancies and births.

Muslim women had their first births earlier than orthodox women. This might be due to normative pressure and traditional cultures of Muslims influence women not to use a contraceptive. It is supported with 68% of Muslims were not used any form contraceptive compared to only 47% orthodoxies were not used in 2016, EDHS [44]. The other justification could be the ignorance of Muslim women. It is also supported by the EDHS report that more than 90% of Muslim women were had primary and no education level [44]. This finding was in concurrence with findings in Bangladesh [23] and Nigeria [25] where 90% and 50% of the population were Muslim religion followers.

The study findings should be interpreted in light of numerous limitations. First, the analysis is based on self-reported information and thus is subject to self-report bias (recall and social desirability bias). For example, there is possibly under-reporting of births ended with death. The choice of predictors in the analysis was also limited to background characteristics. There was the possibility, that other variables not included in the analysis significantly affect time to first birth like parental education and economic status. Some variables like religion are time varying to predict the outcome since current religion only considered in the study.

These limitations notwithstanding, the findings highlight some key factors that are likely to be significant drivers of early entry into motherhood and in a far advanced age at first birth among reproductive-age women in Ethiopia. A key strength of this study is the use of a nationally representative and population-based data to model the timing of first births among reproductive-age women in Ethiopia which make the result to be generalized to the reproductive age women in Ethiopia and similar developing countries.

Another important strength of the finding was accounting for the contextual effect which helps to design strategies for context-based interventions. In statistical analysis the possible suitable model for the data considered.

## Conclusion

In this study, the median age at first birth was found to be 20 years which was at a lower boundary of the optimum age to first birth 20–29 years. Early age at first marriage, sexual intercourse, high spousal age difference, and being Muslim religion followers were predictors of first birth at an early age. On the other hand secondary and higher education levels, living in the most urban regions, contraceptive use were factors to delay first birth.

Early childbirth, which was often originated from early marriage and sexual behavior, result in potential health risks for the young mother and their child, as well as the termination of education and blurred future job prospects. Therefore we recommend: the ministry of women and children affairs’ to introduce programs aiming to reduce early sexual intercourse very early, and before the commencement of the sexual activity.

Better to avoid marriage at an early age and high spousal age difference by teaching the community and enforcing legal marriage age. The Ministry of Education recommended retaining women to at least a secondary education level and higher by extending the accesses to rural dominant and pastoralist regions. The Ministry of Health also better to maximize utilization of contraceptives by increasing access and promoting friendly methods as well.

Researchers better to conduct researches incorporating family factors and investigate what factors may influence in Muslim religion followers to give first birth at an early age.

Better to explore factor associated with delayed first birth among urban region residents and those had secondary and higher education.

## Data Availability

The data underlying the study can be accessed after legal registration at www.measuredhs.com. After registration, interested researchers to conduct study can log in at https://www.dhsprogram.com/data/dataset_admin/login_main.cfm and access the data as zipped files.

## Abbreviations

AHR: Adjusted hazard ratio
AIC: Akaike information criterion
ATR: Adjusted time ratio
CSA: Central statistics agency
CSPro: Census survey program
EAs: Enumeration areas
EDHS: Ethiopian demographic health survey
GG: Generalized gamma
IW: Inverse Weibull
IQR: Interquartile range
MHR: Median hazard ratio
PH: Proportional hazard
PO: Proportional odds
WHO: World Health Organization
